# A Novel Paradigm for Non-Invasive Prenatal Genetic Screening: Trophoblast Retrieval and Isolation from the Cervix (TRIC)

**DOI:** 10.3390/diagnostics13152532

**Published:** 2023-07-30

**Authors:** Kirim Hong, Hee Jin Park, Hee Yeon Jang, Sung Han Shim, Yoon Jang, Soo Hyun Kim, Dong Hyun Cha

**Affiliations:** 1 CHA Gangnam Medical Center, Department of Obstetrics and Gynecology, CHA University, Seoul 06125, Republic of Korea; rachelkh@chamc.co.kr (K.H.); coolsome72@chamc.co.kr (H.J.P.); yoonjang.yj@gmail.com (Y.J.); 2 Department of Biomedical Science, College of Life Science, CHA University, Seongnam 13488, Republic of Korea; jhyeon@chamc.co.kr (H.Y.J.); shshim_ce@chamc.co.kr (S.H.S.)

**Keywords:** trophoblasts, prenatal screening, non-invasive prenatal testing, NIPT, prenatal diagnosis, trophoblasts retrieval and isolation from the cervix (TRIC), transcervical sampling

## Abstract

As the prevalence of pregnancies with advanced maternal age increases, the risk of fetal chromosomal abnormalities is on the rise. Therefore, prenatal genetic screening and diagnosis have become essential elements in contemporary obstetrical care. Trophoblast retrieval and isolation from the cervix (TRIC) is a non-invasive procedure that can be utilized for prenatal genetic diagnosis. The method involves the isolation of fetal cells (extravillous trophoblasts) by transcervical sampling; along with its non-invasiveness, TRIC exhibits many other advantages such as its usefulness in early pregnancy at 5 weeks of gestation, and no interference by various fetal and maternal factors. Moreover, the trophoblast yields from TRIC can provide valuable information about obstetrical complications related to abnormal placentation even before clinical symptoms arise. The standardization of this clinical tool is still under investigation, and the upcoming advancements in TRIC are expected to meet the increasing need for a safe and accurate option for prenatal diagnosis.

## 1. Introduction

One of the latest phenomena in current obstetrics is advancing maternal age. Recent data from the Centers for Disease Control and Prevention (CDC) emphasized the rising trend in the average age of pregnant women in the United States in which almost 19% of all pregnancies were in maternal age of 35 years and older [1]. This trend is observed globally, especially with women’s age at their first pregnancy continuously advancing at a fast pace. In particular, South Korea has drawn attention to its fast change in the average age of first childbirth increasing from 26 in 1993 to 32 in 2020 [2]. Therefore, recent obstetrical consensus had repeatedly addressed major complications in pregnancies with advancing maternal age, including increased risk of fetal aneuploidy which is mentioned with the greatest emphasis [3,4]. The Obstetrical Care Consensus statement in 2022 for “pregnancy at age 35 years or older” by the American College of Obstetricians and Gynecologists and Society for Maternal-Fetal Medicine states that prenatal genetic screening and diagnostic testing options should be offered with detailed counseling based on each patient’s individual risk [5]. Therefore, prenatal genetic counseling has become one of the most imperative tasks for a clinician in current obstetrics.

At present, prenatal genetic screening and diagnostic testing are two separate categories that can be characterized by their invasiveness. Prenatal genetic screening tests are ”non-invasive” methods that include the maternal serum screening of markers related to aneuploidy with or without nuchal translucency ultrasonography, and cell-free DNA screening, known as non-invasive prenatal testing (NIPT). These tests are performed by drawing maternal peripheral blood sampling; therefore, there is no risk of procedure-related pregnancy complications. However, since these tests are only for screening purposes, when the results show a high risk of aneuploidy, confirmative diagnostic testing is mandatory. The diagnostic tests include chorionic villus sampling (CVS) and amniocentesis which are ”invasive”. Since the tests require a needle puncturing through the uterine wall to the actual placenta or amniotic fluid, the risks of complication exist with a fetal loss rate of 0.7% and 0.6% for CVS and amniocentesis, respectively [6].

Therefore, pregnant women tend to prefer non-invasive prenatal screening to invasive diagnostic testing which has led to the rapid spread of NIPT around the world ever since its commercial introduction in 2011 [7]. NIPT is now available in more than 60 countries and the annual growth rate of NIPT has been estimated to be about 10.9% to 17.15% [7]. The estimated global market value for NIPT ranged from USD 2.8 to 3.9 billion in 2019–2020 with the United States accounting for the biggest market share [8]. However, various limitations of NIPT have been continuously raised which have restricted its use as a prenatal genetic test for screening purposes only. Thus, NIPT is often referred to as “NIPS” (non-invasive prenatal screening) since pregnant women can often be confused by the concept of ”screening” and ”diagnostic” and the name of NIPT itself can be misleading [9].

Birth defects related to chromosomal abnormalities can impact personal and family life, both emotionally and financially. Early detection of fetal chromosomal abnormalities can inform expectant parents about congenital disabilities and provide help in making crucial decisions to control pregnancy and childbearing [10]. In this review, we aim to introduce a different, innovative non-invasive prenatal genetic test with a diagnostic value called TRIC (trophoblast retrieval and isolation from the cervix) which allows for obtaining fetal DNA in whole cells by simple cervical brushing, as early as from 5 weeks of gestation.

## 2. Cell-Free Fetal DNA (cffDNA) Analysis Methods

### 2.1. Cell-Free DNA Fragments

NIPT analyzes cell-free DNA (cfDNA) in maternal blood to determine the risk of fetal aneuploidy. The cfDNA is a mixture of predominant maternal DNA originating from the hematopoietic system [11] and fetal DNA molecules, which originate from apoptosis of placental cytotrophoblasts [12,13]. The cell-free fetal DNA (cffDNA) can be detected as early as from 4 weeks of gestation in a highly fragmented form [14,15]. The average length of cffDNA fragments has been reported to be approximately 143 base pairs, and maternal cfDNA approximately 166 base pairs, as a consequence of fragmentation [16]. Fetal-specific preferred end sites were mostly located at the border or within the nucleosome core while the maternal-specific end sites were mostly located in the linker region [17]. Until recently, most studies have concentrated on analyzing short DNA strands, usually less than 500 base pairs. However, Yu et al. reported that longer fragments of cfDNA, measuring up to 23,635 base pairs, can be identified in maternal plasma through single-molecule methylation analysis using long-read sequencing technologies. The proportions of long cell-free DNA molecules in maternal plasma over 500 base pairs were 15.5%, 19.8%, and 32.3% for the first, second, and third trimesters, respectively [18]. The fetal fraction (FF) is the proportion of cffDNA in maternal plasma in relation to the total circulating free DNA which affects the test’s sensitivity. On average, the fetal fraction (FF) of DNA is only around 10.0% with a range of 6.0% to 20% [19]. Therefore, efficient cffDNA extraction and enrichment techniques are important for the accuracy of most analysis techniques. NIPT is a group of tests that utilize the analysis of cell-free DNA fragments in maternal plasma to screen for fetuses affected by common trisomies (trisomy 21, 18, and 13). Some NIPT laboratories provide pieces of information on sex chromosome abnormalities (Turner syndrome, Klinefelter syndrome, XXX, XYY, and various more complex karyotypes), other autosomal aneuploidy, chromosome segmental imbalances (typically, >7 Mb), select microdeletion syndromes, Rhesus blood group typing, and some monogenic disorders [20]. The methods employed in cfDNA targeting, amplification, measurement, and data analysis vary significantly between laboratories.

### 2.2. Massively Parallel Shotgun Sequencing (MPSS)

**Figure 1:**
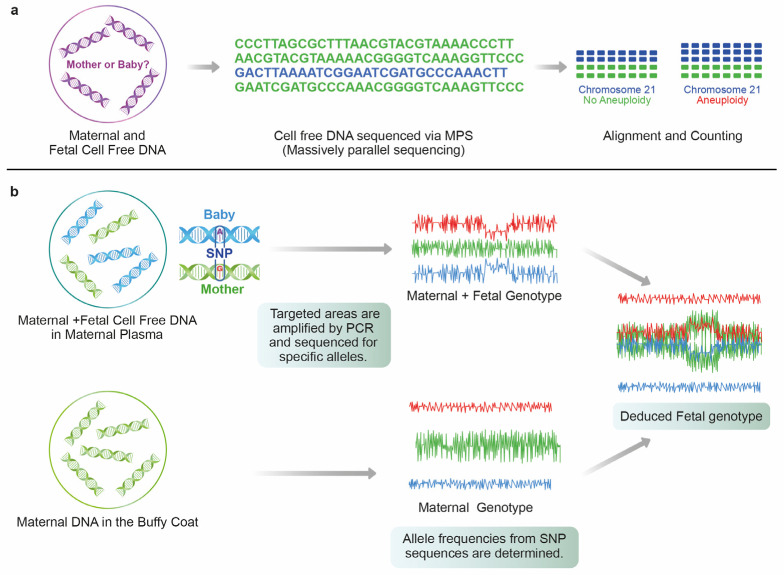
(**a**) MPS-based NIPT (**b**) SNP-based NIPT.

The first report of the detection of trisomic pregnancies utilized massively parallel shotgun sequencing (MPSS) [21,22]. In this method, cfDNA in maternal plasma is sequenced and compared to the human genome to determine its chromosomal origin. As the sequencing process is not selective, millions of DNA fragments from all chromosomes are identified and quantified [23]. As compared to a disomic reference chromosome, MPSS demonstrates the quantitative change in the proportion of each chromosome-derived cffDNA in maternal plasma using next-generation sequencing (NGS), and thus detects fetal chromosome aberrations (Figure 1). In non-pregnant euploid women, around 1.3% of cfDNA is derived from chromosome 21. If both the mother and fetus are euploid, the anticipated percentage of chromosome 21 fragments is 1.3%. In the case of fetal trisomy 21, the proportion of chromosome 21 fragments in maternal plasma cffDNA will be slightly higher. Hence, MPSS discriminates the relative genomic representation of plasma DNA molecules in trisomic pregnancies [24,25]. This technique has a high positive predictive value in identifying ploidy status, especially in trisomy 21 [26]. Although MPSS has shown promising results, its high cost and complexity, including the complexity of data analysis, pose obstacles to widespread clinical adoption. MPSS analyzes random genomic fragments from all chromosomes, leading to large amounts of unutilized sequencing data generation. Subsequently, digital analysis of selected regions (DANSR) was developed where the selective sequencing of loci from chromosomes under investigation is undertaken, thereby increasing throughput, and reducing cost [23]. DANSR requires only 420,000 reads per sample to achieve a similar performance as MPSS, which usually requires 10.8 million sequencing reads per sample. The DANSR mapping specificity of the selected regions’ efficiency was higher than 96%, whilst MPSS mapping rates were up to 50% [27].

### 2.3. Single Nucleotide Polymorphism (SNP)

Another method used for cffDNA analysis is utilizing numerous single nucleotide polymorphisms (SNP) that exist only on the chromosome of interest. A large number of SNP sequences are designed to detect variations on the chromosome of interest. The SNP-based method analyzes polymorphic loci and determines chromosomal copy numbers by looking for specific patterns in allelic distributions (Figure 1). This method does not require a disomic reference chromosome and it is uniquely able to detect the presence of additional fetal haplotypes associated with dizygotic twins and triploidy. The results indicate an increased (or missing) amount of chromosomes in plasma by detecting significant shifts between SNP patterns. The advantage of the use of SNP relies on consistent amplification across alleles at a locus. It can yield consistent copy number calls across chromosomes [23,25,27]. Disadvantages include the inability to offer this procedure to patients who have undergone egg-donated IVF pregnancies or following bone marrow transplantation. Additionally, the SNP method is unable to provide a read when there is a lack of prominent parental heterozygosity as in the case of consanguineous parents [27].

## 3. Limitations of NIPT

In many countries, NIPT is used as a secondary test for women identified as high-risk after the first trimester combined screening, and in others, it is used as a primary screening test. As compared to the prior screening methods for aneuploidy involving sequential maternal serum and/or ultrasound screening, which exhibits trisomy 21 detection rates of 81–96% with a false-positive rate fixed at 5%, NIPT demonstrates higher detection rates for trisomy 21 (99%), and also for trisomy 18 (98%) and trisomy 13 (99%) with much lower false-positive rates of 1–2% [28,29,30,31]. The American College of Medical Genetics now recommends NIPT as a primary screening test for sex chromosome abnormality as well as for fetal trisomies 21, 18, 13 [32].

### 3.1. Fetal Fraction (FF)

However, NIPT exhibits quite a few limitations to consider. First of all, maternal blood contains only a small fraction of fetal cells (1 fetal cell per 10^6^–10^7^ maternal cells) [33]. For the test to be adequately analyzed, a fetal fraction (FF) of cfDNA, which originates from placental cytotrophoblasts in a maternal blood sample, must exceed at least 2–4% of the total cfDNA in the plasma and this can be challenging under various circumstances [34,35]. Numerous factors can result in a low FF either by increasing maternal cfDNA amounts or decreasing fetal (placental) cfDNA concentrations. The most important maternal factor is the maternal body weight; an increased maternal body mass index (BMI) is related to inflammation and necrosis of adipocytes which in turn increases the maternal-derived cfDNA concentrations [36]. Therefore, according to previous study reports, the no-call (unreportable results) rate for NIPT ranges from 5.4% to 70.1% for women with a BMI higher than 40, compared to 0% to 4.2% for women with a BMI between 18.5 and 24.9 [37]. Other maternal factors that can alter the cfDNA concentration include pre-existing maternal diseases, drug use, and assisted reproductive pregnancy [38,39,40].

During pregnancy, women with autoimmune disease may experience a low level of cffDNA as an inflammatory response may cause a rise in maternal cfDNA in the bloodstream. This ultimately leads to a decline in the proportion of cfDNA derived from the fetus. Systemic lupus erythematosus (SLE) patients may exhibit abnormal DNA methylation in their T cells, which, along with T cell apoptosis, can further contribute to an increase in hypomethylated cfDNA in the blood with shorter DNA fragment lengths. Consequently, NIPT analyses using next-generation sequencing may reveal a different pattern in SLE patients compared to healthy patients [41].

**Figure 2:**
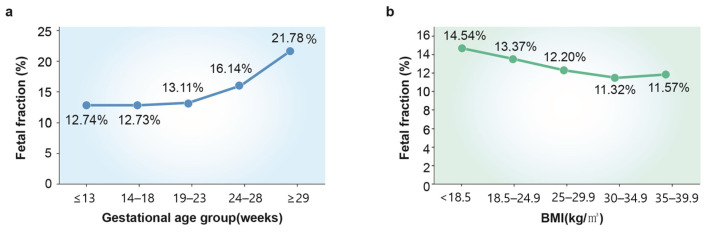
(**a**) Effects of gestational age on the percentage of fetal fraction. (**b**) Effects of maternal BMI on the percentage of fetal fraction. (Data source: Hum. Genomics **2019**, *13*, 62, https://doi.org/10.1186/s40246-019-0244-0 [46]).

Suzumori et al. showed that the concentration of cffDNA decreases in cases of trisomy 13 or 18. The smaller placental size and intrauterine fetal growth retardation observed with trisomy 13 and 18 might contribute to a lower FF [42]. Taglauer et al. reported that fetuses with trisomy 21 have an increased FF when compared to euploid fetuses [12]. This may be reflective of a better test performance for trisomy 21 as compared to trisomy 13 or 18 [43]. The most important fetal factor is the gestational age at the time of blood sampling since the FF increases as the fetus and the placenta grow throughout pregnancy [44]. As of today’s technology, the gestational age should be at least 9–10 weeks for NIPT to be reliably tested in a singleton pregnancy [45]. Hou et al. reported that the percentage of the fetal fraction significantly increased with the increasing gestational age. On the other hand, a decrease in the fetal fraction was observed with an increasing maternal BMI (Figure 2) [46].

### 3.2. Chromosomal Mosaicism

**Figure 3:**
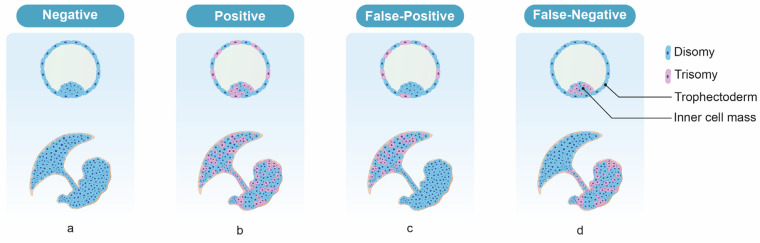
The expected NIPT results according to the types of mosaicism. (**a**) Euploid fetus and placenta, (**b**) generalized mosaicism, (**c**) CPM, (**d**) TFM, in which trisomy cells are absent in the cytotrophoblast but present in the villus mesenchyme and the fetus.

False-positive test results for NIPT may arise due to confined placental mosaicism (CPM) [47]. Since the primary source of “fetal” cfDNA in maternal circulation is placental cells (syncytiotrophoblasts), the cfDNA test is expected to provide results relevant to the placenta, which may be discordant with the actual fetal tissue. Previous CVS cases have shown that discordance may occur in 1–2% of pregnancies [47,48,49,50,51], and is more likely in cases with monosomy X and trisomy 13 than those with trisomy 21 or 18 [52]. True fetal mosaicism (TFM) can also result in false-negative cfDNA results (where the fetus is affected but cfDNA testing indicates no chromosomal abnormality). Although it is quite rare, false negatives in cfDNA results have been reported in Japanese data by a percentage of 0.01% [53]. In these cases, the fetus was chromosomally abnormal but cells with normal karyotype chromosomes existed on the villi [54]. Since the results of NIPT are determined by the relative proportions of cells with normal and abnormal karyotypes within the villi, mosaicism in which a high proportion of cells with normal karyotypes exist may produce a negative result. Since the NIPT result is negative, a definitive diagnostic test is not performed despite the fetus being chromosomally abnormal. In these cases, the cfDNA test results are considered analytically correct (i.e., detecting those placental cells of the mosaicism which are euploid) but clinically incorrect (i.e., the fetus itself is aneuploid) [55]. This kind of situation can occur for trisomy 13 and 18, but not trisomy 21 [56] (Figure 3).

### 3.3. Maternal Malignancies

Chromosomal abnormalities are often present in malignant tumors. If a pregnant woman has a malignant tumor, the NIPT result may be a false positive or may be non-reportable. The risk of confirmed malignancy is significantly higher especially when multiple chromosomal abnormalities are detected by NIPT [55,57]. Catharina et al. reported that a low percentage (0.02%) of NIPT results were assessed as indicative of a maternal malignancy in 231,896 pregnant women [57]. Although relatively rare, malignant tumors occur in about 1 in 1000 pregnant women [58] and account for approximately 15% of false-positive NIPT results [47]. An amniotic fluid examination is performed in NIPT-positive pregnant women spanning multiple chromosomes and if there is no chromosomal abnormality in the fetus, it is necessary to be cautious about the combination of malignant tumors [55]. False positives due to benign tumors such as fibroids have also been reported [59].

### 3.4. Vanishing Twins (VT)

A vanishing twin (VT) is a spontaneous reduction of an embryo and/or gestational sac following documented fetal cardiac activity in both fetuses of a twin gestation during the first trimester [60]. Since chromosomal abnormalities are one of the major causes of miscarriages, trisomies could be the cause of VT, leading to a potentially high number of false-positive results. To avoid inaccurate results, recent guidelines from the American College of Obstetricians and Gynecologists and the Society of Maternal-Fetal Medicine suggest diagnostic testing in multifetal pregnancies if a vanishing twin is identified, instead of relying on serum-based aneuploidy screening or cfDNA [31]. However, other systematic reviews have shown that NIPT can successfully detect common autosomal aneuploidies in pregnancies affected by VT, although with a higher false-positive rate [61].

## 4. Novel Approach for Non-Invasively Retrieving Fetal Cell

Therefore, to overcome the limitations of NIPT, a different method to non-invasively retrieve fetal cells has been constantly investigated. One of the approaches that has drawn attention was obtaining trophoblast cells in the cervix which naturally migrate from the placenta into the reproductive tract [62,63]. Historically, the beginning of this idea originated in the early 1970s when chorionic cells were repeatedly found in human endocervical mucus even in early pregnancy [63]. Although some of the following studies had found contradictory results in replicating the previous findings, doubts were clarified with advancements in fluorescence in situ hybridization (FISH) and PCR techniques [64,65]. Subsequent studies in the 1990s successfully detected fetal trisomy 21 and 18 by FISH from transcervical sampling in mothers of normal karyotype, as well as fetal rhesus-D antigen in rhesus-negative mothers by PCR analysis [66,67,68]. Ever since, continuous efforts have been made by various subsequent studies which focused on effectively sampling and isolating trophoblasts from the cervix, which will be discussed later in detail.

The main mechanism of trophoblast cells traveling down to the cervix had been developed from the fact that upon implantation in early gestation, as the placenta develops by anchoring its villi to the uterine decidua, extravillous trophoblast (EVT) cells are differentiated and they invade into the glands and blood vessels of the uterus [69]. These EVT cells travel along the interstitial, endovascular, and endoglandular routes and are eventually transported into the uterine cavity with glandular secretions toward the cervix where they can be non-invasively retrieved by simple brushing [70].

## 5. Trophoblasts Migrating to the Cervix

Placenta starts developing once a blastocyst implants in the uterine decidua. During the placentation period of 5 to 12 weeks in pregnancy, trophoblasts at the base of the anchoring villi differentiate into EVT cells, and this allows for embedment in the uterus [71]. The migratory characteristics of EVT cells are achieved as villous trophoblasts differentiate into “extravillous” trophoblasts by expressing HLA-G antigen and specific integrin subunits (such as β1) [72,73]. Therefore, HLA-G is a suitable marker of differentiated EVT, used for sorting out EVTs from maternal cells when transcervical sampling is obtained.

There are two different secretion and migration paths of EVTs through the endometrial tract. Interstitial EVT cells penetrate the uterine epithelium at the margin of the placenta and replace the uterine epithelial lining from the basal side, exposing themselves to the uterine cavity [74]. Another route can be endoglandular EVT cells invading uterine glands at the transitional zone of decidua basalis and parietalis. While the placenta grows, the lateral margin continuously exposes new glands for endoglandular EVTs to invade, which could eject EVTs with glandular secretions into the uterine cavity [69]. Once they reach the uterine cavity, EVTs can be transported to the cervix with secretions along the endometrial tract.

Therefore, it is speculated that EVT cells migrate to the cervix only during the limited period of placenta growth. Previous studies were able to successfully obtain EVT cells by cervical brushing as early as from 5 weeks of gestation and only up to 20 weeks of gestation [75,76,77]. Although published results stated that trophoblast retrieval from the cervix was not affected by gestational age between 5 and 20 weeks, further study with a larger sample size is required to confirm the actual range of gestational age that is best suitable for this prenatal diagnosis method (Table 1).

## 6. Technical Development of EVT Cell Isolation and Identification

TRIC was developed as a solution to the technical challenge of analyzing placental cells in real time. The unique expression of HLA-G was found on the surface of EVT cells and by using the antibody to this antigen, adult tissues could easily be excluded from the samples obtained via the reproductive tract, thereby leaving the cell population of our interest [78]. Imudia et al. described the “Trophoblastic Retrieval and isolation of Cervix” (TRIC) method, performed on endocervical samples collected between 5 and 20 weeks of gestation by brush insertion through the external os, approximately 2 cm into the endocervical canal, followed by rotations to trap mucus. The average frequency of HLA-G positive cells in normal intrauterine pregnancy cervical samples was approximately 1 in 2000, which was 4-fold higher than samples from patients with ectopic pregnancy and blighted ovum (*p* < 0.001). This pilot study presents evidence that trophoblast cells can reliably be obtained and identified among cervical cells in the first trimester by immunohistochemical staining for HLA-G and suggests for the first time that abnormal pregnancies may be predictable based on the abundance of trophoblast cells in the cervical canal [79].

A limiting feature of using trophoblast cells obtained from the cervix for non-invasive prenatal testing is the excessive presence of maternal cells which makes fetal cell isolation difficult. TRIC protocol using HLA-G for immunomagnetic isolation has been developed to obtain trophoblast cells from the cervix with a high degree of purity in ongoing pregnancies [77]. Jain et al. obtained an average of 282 intact trophoblast cells with a fetal DNA fraction of 92.2 ± 6.5% by magnetic-activated cell sorting (MACS) using an anti-HLA-G antibody specific for EVTs [80]. Following the original protocol, another study achieved 44% of cases with a high fetal fraction. This was at a much lower success rate compared to the original TRIC study; nonetheless, the number of isolated cells were sufficient with an average number of 700 cells. The likely cause of reduced success compared to the original study is possibly due to more careful sampling. Researchers have suggested that sample collection from the endocervix closer to the exocervix can yield samples with more maternal cell debris excretion that contains greater amounts of free-floating DNA [76]. Pfeiffer et al. used a sampling method of brush rotations at the external part of the endocervix entrance similar to a PAP test. EVT cells were isolated using the “Isolation by Size of Tumor/trophoblastic cells” (ISET) system, comprising sample filtration through an 8 μm pore device. Putative fetal cells were morphologically identified under light microscopy and micro-dissected for genotyping analyses. Trophoblastic cells were recovered from all tested cervical samples with a frequency of 2–12 trophoblasts per 2 mL [81].

Because cell isolation methods based on visual recognition and micromanipulation are time-consuming, and the specificity is operator-dependent, Bourlard et al. tested partially automated methods for EVT cell retrieval. HLA-G positive cells diluted in HLA-G negative cells were isolated by flow cytometry (FACS Aria III) or magnetic cell sorting (DynaMag™-2 Magnet) but no HLA-G positive cells could be recovered from exocervical samples. This result suggested that trophoblasts are too rarely and inconstantly present in non-invasive exocervical samples to be reliably retrieved by standard immunoisolation techniques [82]. Endocervical samples were suggested to provide up to 10-fold more EVT cells (100–1000 cells) [83] compared to exocervical samples (2–60 cells) [81,84], which likely explained the difference between the study by Bourlard et al. [82] and the aforementioned original TRIC study [80] where fetal cells were isolated with a purity of approximately 90%.

## 7. TRIC Methods

**Figure 4:**
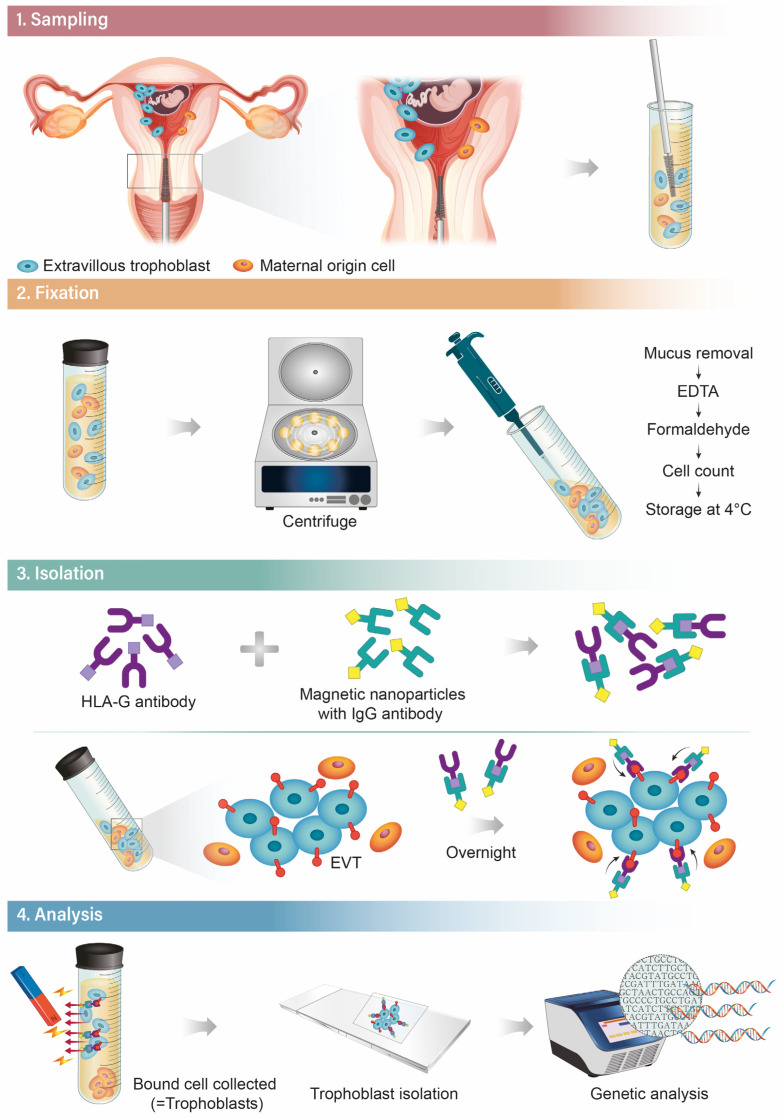
Four steps of TRIC (trophoblast retrieval and isolation from the cervix).

Ever since the first discovery of trophoblasts in the cervix, various studies have proposed methodologies for obtaining the trophoblasts and technical ways for isolating trophoblasts for analysis. This comprehensive method named TRIC (trophoblast retrieval and isolation from the cervix) can be outlined in four steps, as depicted below (Figure 4).

### 7.1. Sampling

Historically, sampling of maternal cervical mucus has been attempted via several different methods. The techniques include endocervical lavage, cervical mucus aspiration, or cytobrushing which all succeeded in obtaining trophoblasts with various success rates [85,86]. As of now, the cytobrush used in the Papanicolaou test is the most effective and easily used for sampling [70]. The patient is put in a lithotomy position, and “endocervical” sampling is performed with a cytobrush—the brush is gently inserted through the external cervical os with a depth up to 2–3 cm. In this inserted position, the brush is rotated in a full circle (360°) to draw sufficient cells. From the authors’ previous experiences, women with active vaginal bleeding should be excluded since it interferes with the procedure due to substantial maternal cell contamination of the sample. Likewise, the operator must be aware of the fact that rough handling of the brush resulting in cervical tissue bleeding can ruin the quality of the sample.

### 7.2. Fixation

After the endocervical sampling, the brush needs to be immediately immersed in phosphate-buffered saline (PBS) solution in a test tube. Then, the sample should be acidified with 3% acetic acid (300 μL/10 mL) at room temperature for 5 min to dissolve mucus products and centrifuged at 900× *g* for 5 min at 4 °C. After washing the cells three times in 20 mL of PBS, the sample is fixed with a formaldehyde 3.7% solution at 4 °C for 10 min. Then, the fixed cells are centrifuged at 900× *g* for 5 min, washed with cold PBS three times, and counted for analysis.

### 7.3. Isolation

Once a successful sampling of the endocervical cells is achieved, another key step is the sorting of fetal cells (EVTs) from maternal cells. In the beginning era of TRIC studies, a micromanipulation isolation technique was used which sorted out cell clumps with the villous morphology of trophoblast cells [87,88]. Recently, a new method has been developed which utilizes the immunomagnetic isolation technique targeting the unique expression of HLA-G in EVTs, henceforth maximizing the purity of the isolation process [70,71,78]. Anti-HLA-G antibodies are incubated with magnetic nanoparticles that are conjugated to a goat anti-mouse immunoglobulin G (IgG) antibody (Figure 1) overnight at 4 °C. The next day, the collected endocervical cells are resuspended in PBS and mixed with the nanoparticle-attached HLA-G antibodies and incubated overnight again at 4 °C. Finally, the magnetic nanoparticle-bound cells, which are assumed to be EVTs, are separated from the non-bound cells (maternal cells) by using magnetic immobilization [89].

### 7.4. Analysis

After the isolation, confirming the purity of EVTs in the final specimen is imperative. Expression levels of b-hcg can be measured as a trophoblast marker by immunocytochemical labeling [77]. Several studies have reported a high percentage of the b-hcg rate after the isolation of up to 95%, but these values show large variations between different study designs and TRIC protocols [70,89]. Other trophoblast markers used include antigens expressed by EVTs and cytokeratins; cytokeratin 7 and placental lactogen (CSH1) are some of the most useful markers that are specifically expressed in trophoblasts [70,90].

FISH can also be useful especially when X and Y chromosome probes are used for analyzing the isolate. Since FISH directly labels specific genes with fluorescent dyes, it enables the visualization of the number of copies of genes or chromosomes of each cell. Therefore, numerous studies have demonstrated XY signals in specimens from male fetuses that validate the existence of fetal-origin cells [70,89,91]. Moreover, FISH and PCR techniques have been applied to determine common aneuploidies in transcervical specimens, as well as fetal hemoglobin genotypes related to thalassemia and sickle cell disease [92,93,94].

A recent experiment has accomplished the DNA profiling of EVTs obtained by TRIC by the next-generation sequencing of SNPs and STRs. The study collected samples at 5 to 19 weeks of gestation and fetal DNA fractions were 85 to 99%, with 100% concordance between the allelic profiles of fetal DNA and reference placental DNA. It also showed 100% correct fetal gender identification in all samples [80]. Although a follow-up study with the same protocol reported a much lower rate of success in fetal DNA analysis at 23% to 44%, the expectation remains high since it has shown the possibility for non-invasively detecting a single gene disorder as early as from 5 weeks of gestation [76].

## 8. Refining Updates for TRIC Techniques

Despite copious preceding studies, the reproducibility of TRIC remains controversial due to its varying success rates (75–100%) reported in confirming fetal cells [78]. Since this limitation can hinder the clinical application of TRIC as a prenatal diagnostic tool, it is crucial to establish the most accurate and reproducible techniques that achieve consistent results in any clinical setting.

One of the most important steps in TRIC is fixation. Previously, our institution used an alcohol-based Thinprep solution composed of water (40–70%) and methanol (30–60%) for the sample fixation [81,85]; this solution had been chosen since the sampling procedure had been performed concurrently with the routine Pap screening in early pregnancy. However, upon continuous sample collections at our institution, we figured out that as soon as the endocervical sample (cytobrush) is immersed in the Thinprep solution, cells would rapidly aggregate (probably due to the alcohol components in the solution) and often result in failure in further analysis. Therefore, we have designed a new method of fixation called “post-fixation” which involves the rapid immersion of the sampled brush in PBS. The maternal cells are then removed by acetic acid and centrifugation, followed by the fixation of the remaining cells in a formalin solution [75]. Several obtained fetal cells were compared between the two methods: pre-fixation vs. post-fixation. The post-fixation method demonstrated much less cell aggregation and resulted in a statistically significant increase in the percentage of b-hcg positive cells (83.2 ± 8.1% compared to 66.4 ± 13.3%, *p* = 0.003) [75]. This study suggested that although the traditional pre-fixation method could be convenient, the post-fixation method should be used to maximize the accuracy of the fixation protocol.

Another key technique in TRIC is the immunomagnetic isolation of the trophoblasts. To maximize the purity of captured fetal origin cells (EVTs), the antibody that binds to the antigen specifically expressed only in EVTs should be chosen. As mentioned earlier, HLA-G expression is one of the characteristic features in EVTs which distinguishes them from maternal-origin cells. Different types of HLA-G antibodies such as G233 and 4H84 are known to bind to them [95]. A recent study at our institution aimed to compare the effectiveness of trophoblast isolation using two different HLA-G antibodies.

HLA-G structurally consists of heavy-chained alpha domains (α1, α2, α3) and β2-microglobulin in extracellular regions which results in seven isoforms (HLA-G1 to G7) based on different combinations of the domains [96]. Each HLA-G antibody can bind to different isoforms of HLA-G. The main difference between G233 and 4H84 is that G233 only binds to the native HLA-G1 isoform while 4H84 binds to the HLA-G1 and HLA-G2 isoforms [97]. We believed that this difference can significantly affect the efficacy of the immunomagnetic isolation step in TRIC since HLA-G2 expression is exclusively shown in invasive trophoblasts (EVTs) while HLA-G1 is abundantly expressed in many other trophoblast cell subpopulations. As expected, the study demonstrated that the TRIC technique using the 4H84 HLA-G antibody increased the purity of b-hcg-expressing cells as compared to using G233 with a statistical significance (82.6 ± 7.1% vs. 62.4 ± 8.24%, *p* < 0.001) [89].

Nonetheless, the immunofluorescence results of these filtered HLA-G immunoisolated trophoblasts showed interference with maternal mononuclear cells that are retrieved during sample collection. Previous immunophenotyping studies have proved CD56 expressions on the surface of maternal mononuclear cells [98,99], and by using these expressions as a biomarker, an immunomagnetic exclusion of uterine NK cells can be performed. In an additional immunomagnetic fetal cell inclusion filtration, a double-step isolation procedure can help obtain further purified fetal trophoblastic cells. In a further study, we have selected a specific surface marker to remove maternal immune cells. The modified method of TRIC was suggested to increase the purity of the trophoblast by adding a procedure removing mononuclear cells via MACS. Overall, these delicate advancements in TRIC techniques are expected to refine the accuracy of TRIC to become the next revolutionary non-invasive prenatal diagnostic test.

## 9. Future Expectations

Previous large population-based studies analyzing the effectiveness of primary screening through cfNIPT have shown that cfNIPT had a miss-detection rate of 17–25% of atypical fetal chromosomal aberrations [100,101,102,103]. If enough trophoblast cells are isolated during the TRIC procedure, every cell carries the potential for an entire fetal genome uncontaminated by maternal DNA. Thus, they may constitute an attractive source for non-invasive prenatal testing not only for common trisomies but also for atypical chromosome aberrations such as triploidy, deletion, duplication, unbalanced structural rearrangement, mosaicism, rare autosomal trisomy, confined placental mosaicism (CPM), and more. A recent meta-analysis showed that the risk of delivering small-for-gestational-age neonates (<10th centiles) was 3-fold higher for confined placental mosaicism excluding trisomy 16, and 11-fold higher for cases including trisomy 16 only vs. unaffected controls, respectively. CPM resulted in a much higher risk of birthweight below the 3rd percentile (odds ratio, 5.33). Robust evidence suggests an increased risk of impaired fetal growth irrespective of prematurity in pregnancies with CPM, suggesting the need for closer antenatal surveillance. This study confirmed that CPM showed the theoretical potential for being an etiological precursor of placental dysfunction [104]. Once established as a standardized protocol, EVTs collected via TRIC could provide fetal genetic information at a similar quality to that of chorionic villus sampling (CVS). TRIC may detect placental mosaicism in early pregnancy non-invasively. Henceforth, adopting TRIC could provide patients with CPM with an option to choose amniocentesis over invasive CVS.

A recent follow-up study on TRIC by another institution reported that 44% of samples had an adequately high fetal DNA fraction for genetic testing (which is defined as less than 20% of maternal contamination), and in 23% of cases, a single nucleotide variant (SNV) carried by the unborn fetus was correctly identified by Sanger sequencing [76]. Nonetheless, the authors pointed out that the achieved success rate was lower than the results reported by the original TRIC study by Jain et al.—which demonstrated 85 to 99.9% of fetal DNA fractions with 100% correct haplotyping. The reasons for such disparities in fetal cell purity could be due to various confounding factors such as sample collecting methods, experimenters’ variability, gestational age, isolation techniques, and various clinical statuses of pregnancy which could affect the results [80].

Pregnancy-associated complications that are related to placentation can affect the number of cells obtained by TRIC. This can be expected intuitively since TRIC targets the retrieval of EVTs which are shed from the placental margin as the placental bed grows in size with embedding vascularization. Studies have demonstrated that the rate of HLA-G positive cells obtained by TRIC was significantly lower (by about 4-folds) in abnormal pregnancies, such as ectopic pregnancy and blighted ovum, compared to normal intrauterine pregnancies (*p* < 0.001); in other words, early pregnancy failures such as ectopic pregnancy and blighted ovum can be distinguished from normal pregnancies with a 97% positive predictive value, 87% negative predictive value, 93% sensitivity, and 95% specificity [79]. This study provided a shred of evidence that TRIC can be further developed as a non-invasive approach that could predict early pregnancy loss.

Moreover, further serious obstetrical complications related to placentation in advanced gestation are intrauterine growth restriction (IUGR) and preeclampsia. The failure of EVT cells to invade uterine spiral arteries and to remodel the maternal vascular system is a key contributing factor leading to poor placentation [105]. Therefore, several studies have implied that HLA-G expression is decreased in preeclamptic placentas in comparison with normal placentas [106,107]. This can also affect the abundance of EVTs isolated by TRIC since it utilizes immunomagnetic isolation by HLA-G antibodies. Fritz, R. et al. experimented on comparing the trophoblast yield by TRIC based on various pregnancy outcomes; as expected, in pregnancy complications related to abnormal placentation—which include early pregnancy loss, preeclampsia, and intrauterine growth restriction—the number of obtained trophoblasts decreased although it could not reach statistical significance (510 (IQR 250–786) vs. 750 (IQR 400–1020, *p* = 0.052)) [77].

Furthermore, another study was designed to evaluate the expression of particular proteins known to be related to abnormal placentation in EVT cells retrieved by TRIC. This was to determine whether the expression levels in cases who later develop IUGR or preeclampsia are different from those in normal pregnancies. The transcervical samples were collected between 6 and 20 weeks of gestation and the purity rate of isolated fetal cells was found to be 97.7% in the control groups and 96.6% in the adverse outcome group without statistical significance. The results showed that in cases of IUGR and preeclampsia, the expression of pregnancy-associated plasma protein-A (PAPPA), soluble fms-like tyrosine kinase-1 (sFLT1), soluble endoglin (sENG), alpha-fetoprotein (AFP), placental growth factor (PGF), and galectin 14 (LGALS14) were already altered in EVT cells obtained before 20 weeks of pregnancy with statistical significance. Specifically, sFLT1 and sENG (anti-angiogenic factors) were increased and PGF (a pro-angiogenic factor) was decreased in the adverse outcome group [108]. These results are particularly significant since it is already known that sFLT, sENG, and PGF are reliable biomarkers that are altered in maternal serum several weeks before clinical signs of preeclampsia develop [109,110]. In fact, in current practice, obstetricians use the sFlt-1/PlGF ratio in maternal blood as a useful clinical tool to rule out preeclampsia development within one week [111,112].

Overall, these studies imply the infinite potential of TRIC which can provide valuable information about uteroplacental insufficiency even before clinical symptoms develop. Compared to the currently used clinical tools, TRIC will have a greater advantage in its usefulness in early pregnancy. Continuous investigations about trophoblasts obtained in the cervix by TRIC are required to develop a novel non-invasive technique that can accurately predict placenta-related pregnancy complications.

## 10. Conclusions

Providing professional prenatal genetic counseling to pregnant women with options for various prenatal genetic testing is crucial in obstetrics. Currently, NIPT (cell-free fetal DNA testing) is most widely used as a prenatal genetic screening analysis, but it has its limitations as it requires confirmation via other invasive tests. On the other hand, TRIC is a promising non-invasive diagnostic tool that directly obtains intact fetal cells from the maternal cervix. In addition to its non-invasiveness, TRIC exhibits many other advantages such as possible early detection at 5 weeks of gestation, and consistent results independent from maternal obesity. Moreover, the trophoblast yields from TRIC can provide valuable information about obstetrical complications related to abnormal placentation even before clinical symptoms arise.

Numerous studies to date have built a strong foundation for the future validation of TRIC. Over the next few years, additional studies are expected to establish a thorough method that can be applied in the actual clinical setting as a diagnostic tool for prenatal testing. Future study targets may be suggested to investigate negative selection methods to remove maternal cells from transcervical samples for a maximum purity rate and to elaborate on DNA extraction or analysis techniques for precise diagnosis. Hopefully, these advancements could meet the globally enlarged need for safe, new prenatal diagnosis options.

## Figures and Tables

**Table 1:** Comparison of NIPT vs. TRIC in prenatal genetic testing.

	NIPT	TRIC
Invasiveness	Non-invasive	Non-invasive
Approach method	Maternal serum	Maternal cervix brushing
Targeted fetal genetic source	Cell-free DNA	Extravillous trophoblast
Obtained fetal DNA	Fragmented	Whole
Gestational age	≥9 weeks	≥5 weeks
Maternal BMI, gestational age	Affected	Not affected
Purpose	Screening only	Can be diagnostic

## Data Availability

Not applicable.
